# Opportunities and Challenges of mRNA and VLP Technologies for Pan-Flavivirus Vaccine Development: Focus on Conserved Quaternary Epitope Conformations

**DOI:** 10.3390/ijms27021081

**Published:** 2026-01-21

**Authors:** Eduar Fernando Pinzon Burgos, Sigrid Camacho Ortega, Ben Atkinson, Joel V. Chua, Alonso Heredia

**Affiliations:** Division of Clinical Care and Research, Institute of Human Virology, University of Maryland School of Medicine, Baltimore, MD 21201, USA; epinzonburgos@ihv.umaryland.edu (E.F.P.B.); scortega@ihv.umaryland.edu (S.C.O.); benatkinson26@gmail.com (B.A.); jchua@ihv.umaryland.edu (J.V.C.)

**Keywords:** pan-flavivirus vaccine, mRNA vaccine, VLP vaccine, quaternary epitope

## Abstract

Mosquito-borne flaviviruses, including Dengue virus (DENV), Japanese encephalitis virus (JEV), West Nile virus (WNV), Yellow fever virus (YFV), and Zika virus (ZIKV), continue to present a significant threat to public health worldwide. In 2024, these viruses accounted for 11,717 reported cases in the United States and more than 7.6 million cases globally. As of early 2025, according to CDC data, 1830 cases of dengue had already been reported, with 1584 transmitted locally within the U.S. Despite the considerable burden that these diseases pose, no specific antiviral treatments exist. A very limited number of virus-specific vaccines have been licensed, such as those for YFV, JEV, and, with specific constraints, for DENV. To date, no pan-flavivirus vaccine is available. This review examines the potential of emerging vaccine platforms—particularly messenger RNA and virus-like particles—as promising tools in the pursuit of a broadly protective flavivirus vaccine. We analyze current strategies for inducing cross-neutralizing immune responses and discuss how these technologies could support the presentation of conserved quaternary epitope conformations, which are increasingly recognized as critical targets for establishing potent immune responses. We review key advances in virology, immune response, and immunogen delivery systems to highlight the potential for developing a pan-flavivirus vaccine.

## 1. Introduction

Flaviviruses are enveloped, positive-sense, single-stranded RNA viruses from the *Flaviviridae* (genus *Flavivirus*) family [1]. Flaviviruses can be spread by many arthropod vectors, such as ticks and mosquitoes, making them a threat to much of the world’s population. According to The Global Burden of Disease, an estimated 59.2 million people became infected with dengue virus (DENV), yellow fever virus (YFV), or Zika virus (ZIKV) worldwide in 2021 [2]. Then, in 2024, an epidemic of dengue resulted in more than 13 million cases across North, Central, and South America alone, prompting the Center for Disease Control and Prevention (CDC) to issue a Health Alert about the ongoing risk of DENV in the United States in March of 2025 [3].

Although significant effort has been made in developing vaccines for individual flaviviruses, a pan-flavivirus vaccine is not currently available. Therefore, only virus-specific vaccines are available for some flaviviruses, some of which raise concerns about safety, efficacy, and proper population vaccination coverage [4,5].

For instance, the long-term efficacy and safety of dengue vaccines in endemic regions remain largely unknown [6]. Safety concerns were raised about the first dengue vaccine (Dengvaxia^®^) when dengue naïve vaccine recipients experienced increased rates of hospitalization and severe dengue [7], as well as antibody-dependent enhancement (ADE) [8].

ADE is an adverse immunological phenomenon described mainly during a secondary heterologous flavivirus infection, which causes increased viral replication and worsening of the disease [9]. To overcome these issues, new vaccines are being developed. Qdenga, developed by Takeda Pharmaceuticals, has received attention because of its high efficacy against virologically confirmed cases [10]. However, concerns have been raised about vaccine-induced disease enhancement due to the generation of non-neutralizing antibodies and dengue serotype specificity [11].

Serotype specificity is also a concern in another vaccine recently tested, Butantan-DV, developed by the National Institutes of Health (NIH) [12]. Butantan-DV protected against DENV-1 and DENV-2. Yet, vaccine efficacy against DENV-3 or DENV-4 is unknown because of the lack of these two serotypes in field trials [13].

Despite having a highly effective YFV vaccine as early as the 1930s, yellow fever outbreaks continue to occur [14]. From December 2024 until April 2025, 212 cases have been confirmed in South America, of which 85 deaths have been reported [15]. This is partly due to resource limitations, low vaccination rates in endemic countries [16], population encroachment into YFV-endemic regions [17], and climate change [18]. The live attenuated YF-17D vaccine represents a historic success in flavivirus vaccinology, providing long-lasting immunity to YFV with a single dose [19]. However, its use is contraindicated in the immunocompromised populations, including infants and the elderly, due to rare but potentially severe vaccine-associated viscerotropic and neurotropic disease [20]. Moreover, YF-17D vaccination could enhance DENV infection in certain individuals [21], raising additional concerns about potential immune interference and diagnostic challenges in co-endemic settings [22].

Recognized as a Public Health Emergency of International Concern in 2016 by the World Health Organization (WHO) [23]. Zika remains a public health threat for the Americas, with over 44,000 reported cases in 2024 [24]. Several vaccine candidates have been developed, but none have been licensed [25,26,27]. Concerns have also been raised about the potential of enhancing infection via vaccine-mediated ADE, worsening disease from other flaviviruses (e.g., DENV). In vitro studies have shown that ZIKV can generate poorly neutralizing cross-reactive antibodies targeting the highly conserved fusion loop (FL) in domain II of the viral E protein (DII), which may enhance DENV infection [28,29].

Similarly, several Japanese encephalitis virus (JEV) vaccines, including inactivated and live attenuated formulations, have been deployed with significant benefits to public health. Before the widespread use of vaccines, more than 1.4 million cases of JEV were reported in East Asia [30]. For instance, in China, morbidity from JEV was reduced by 97% from 1971 to 2005, thanks to the introduction of the vaccines. Similarly, JEV vaccinations in Nepal, Sri Lanka, and Malaysia have been associated with a reduction of approximately 72% [31], 95%, and 73% in JEV cases, respectively [32]. Nonetheless, protection remains virus-specific and does not extend to other flaviviruses. Moreover, in some contexts, this virus-specific protection could potentially enhance ADE, limiting its utility in regions with co-circulation of multiple flaviviruses [33]. Furthermore, waning immunity and the need for boosters have been reported with some inactivated formulations [34].

On the other hand, West Nile Virus (WNV), another neurotropic flavivirus, has become endemic in North America, southern Europe, and parts of Africa and Asia [35,36]. While veterinary vaccines exist for equine use [37,38], no human vaccine is currently licensed, despite several candidates reaching clinical trial phases involving the administration of multiple doses and boosters [39,40]. The development of a human WNV vaccine has been slowed by the unpredictable nature of outbreaks, the sporadic distribution of disease, and the difficulty in conducting efficacy trials [41]. The possibility of cross-reactive non-neutralizing immune responses—as seen with other flaviviruses—the potential of ADE or immune interference presents a complex scenario that needs to be addressed. As such, definitive clinical evidence for these effects remains limited [42].

Collectively, the limitations of current flavivirus vaccine products underscore key immunological obstacles, including serotype specificity, cross-reactivity, epitope dominance, and the risk of vaccine-enhanced disease through ADE. These challenges highlight the urgent need for novel vaccine products that elicit balanced, durable, and safe immune responses across flavivirus species and serotypes.

Cryo-electron microscopy of mature viral particles has confirmed the similarity between the structures of the viral E proteins in ZIKV, DENV, and WNV [43]. However, vaccine efforts have largely focused on monovalent strategies, targeting monomeric epitopes, with limited success in eliciting broad protective immunity across multiple serotypes or species. These challenges have stimulated a recent shift in focus from linear or monomeric epitopes to conformational and quaternary epitopes, which more accurately reflect the antigenic surfaces presented on native virions and are increasingly recognized as critical for eliciting broadly neutralizing antibodies (bNAbs) [44,45].

Recent advances in B-cell repertoire analysis and structural virology of the flaviviruses have identified several human monoclonal antibodies (mAb) that recognize quaternary structure-dependent epitopes and have a superior neutralizing activity. For instance, 1C19, a DENV-specific mAb, can bind to a quaternary epitope at the Domain II fusion loop, which is a readily accessible site at the exposed surface of the virion’s E protein of all four DENV serotypes. Importantly, 1C19 does not induce ADE [46]. Other mAbs (EDE1 and EDE2) also neutralized both ZIKV and DENV [47].

More recently, E dimers were arranged in authentic quaternary conformations. Delivery of these quaternary conformations with a virus-like particle (VLP), which mimics the architecture of the native virion but lacks infectious viral RNA, effectively protected mice from ZIKV infection [48].

The term VLP denotes a molecular assembly that contains structural proteins of a virus of interest—such as capsid, core, or envelope proteins—that closely recapitulate the molecular composition and morphological characteristics of the original virus particle [49]. Due to the absence of viral nucleic acids, VLPs are non-replicative and non-infectious, providing a robust platform for vaccine development and immunological studies [50]. VLPs can be engineered from a wide range of viruses by cloning the genes encoding the relevant structural proteins into an expression vector. The construct is introduced—via transfection or transformation—into a suitable host expression system, such as mammalian, insect, or bacterial cells. Within these cells, the transcribed and translated proteins may undergo proper folding and spontaneous self-assembly, resulting in the formation of VLPs [49,51].

VLP vaccines induce broadly neutralizing responses in murine and non-human primate models of ZIKV infection, eliciting high virus-neutralizing antibody titers [52]. On the other hand, mRNA vaccines also present a promising strategy for developing a flavivirus vaccine.

mRNA vaccines are a nucleic acid-based immunization platform that delivers synthetic mRNA encoding a protein of interest into host cells, where it is translated to produce the encoded viral protein [53]. The synthesis and presentation of this protein of interest induces both humoral and cellular immune responses, establishing immunological memory without exposure to a live pathogen [54].

In this regard, VanBlargan and colleagues successfully protected mice from infection with Powassan virus, a tick-borne flavivirus, using a lipid nanoparticle (LNP) -mRNA vaccine [55]. Interestingly, this mRNA vaccine induced cross-neutralization antibodies against other tick-borne flaviviruses, providing an example for the utility of this platform for developing vaccines against several flaviviruses [55].

The identification and use of quaternary structural epitopes represent a breakthrough in the rational design of broadly protective flavivirus vaccines. This review evaluates the potential of virus-like particle (VLP) and mRNA vaccine platforms for the development of a universal pan-flavivirus vaccine based on conserved quaternary epitopes shared across flaviviruses. It examines strategies to induce broadly neutralizing immune responses capable of overcoming viral diversity and minimizing the risk of ADE, and merges current knowledge of vaccine approaches for flavivirus with cutting-edge technologies.

## 2. The Structural Biology of Flavivirus Envelope Proteins

### 2.1. E Protein Domains (DI-DII, DIII)

**Figure 1:**
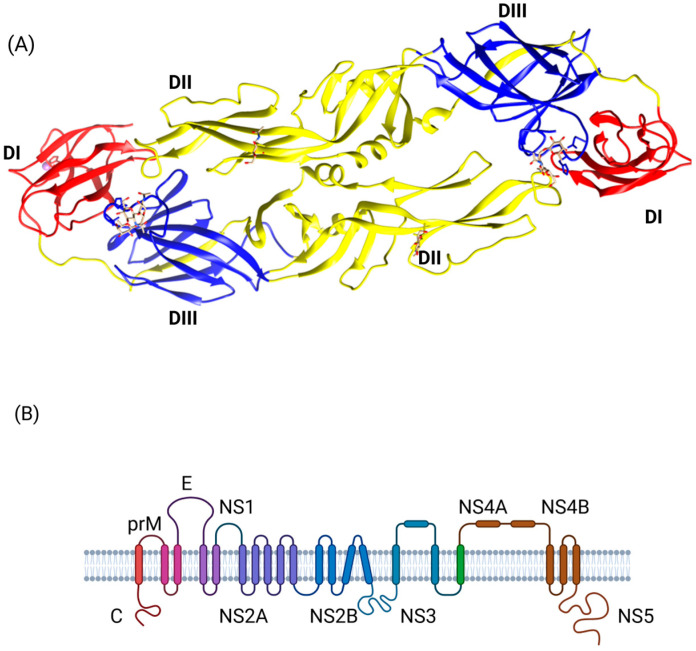
Structure of the Flavivirus E protein. (**A**) The DENV-2 Envelope Protein structure (PDB ID:10AN) was obtained from the RCSB Protein Data Bank [60] and visualized using UCSF Chimera, developed by the Resource for Biocomputing, Visualization, and Informatics at the University of California, San Francisco, with support from NIH P41-GM103311. Flavivirus E protein showing the 3 domains: DI, DII, and DIII in red, yellow, and blue, respectively. Created in BioRender. Pinzon, E. (2025) https://app.biorender.com/illustrations/694dea0185f314172db2a044. (**B**) Polyprotein and transmembrane domain of Flaviviruses. Created in BioRender. Pinzon E (2025) https://app.biorender.com/illustrations/695196786834159ac0d35c6f.

Flaviviruses have a genome of 10.7 kb transcribed into a single polyprotein precursor encoding three structural proteins and seven non-structural proteins [56,57]. The three structural proteins are the Capsid, Premembrane (prM), and Envelope (E) (Figure 1). The E protein is the primary antigen that induces immunity and mediates entry into cells through both binding [58] and fusion [59].

The E protein is an elongated protein with a length of ~ 170 Å and a weight of 60 kDa. Crystallization of the protein revealed three domains: I (DI), II (DII), and III (DIII) [60]. E protein exists as homodimers expressed on the viral membrane of the mature virus, arranged as 30 “rafts” and organized into a herringbone pattern [61].

DI is an eight-stranded central β-barrel structure containing 120 residues in three segments (residues1–51, 137–189, and 285–302). The two long loops between these three segments create the dimerization domain DII [62].

DI is located in the middle of the E protein, acting as a link between the other two domains: DII and DIII. It contains predominantly type-specific non-neutralizing epitopes [63]. DI stabilizes the overall orientation of the protein, and the histidine present in the hinges linking DI with the other two domains triggers conformational changes on DII and DIII upon membrane fusion with the host cell [64] (Figure 1).

DII is an elongated dimerization domain containing the highly conserved internal fusion loop involved in membrane interactions during fusion [59]. This domain contains cross-reactive epitopes that elicit both weak neutralizing and non-neutralizing antibodies [63,65]. Sequential alignment of conserved residues with N-linked glycosylation sites has predicted their presence in most flaviviruses. For example, homology models demonstrated that the fusion loop is identical between DENV and ZIKV [66]. Computational analysis revealed that the fusion loop is a highly conserved epitope across the other human flaviviruses [67], including YFV and WNV [68].

Finally, DIII forms a β-barrel structure composed of six to nine antiparallel β-strands resembling the human immunoglobulin constant domain. It contains ~100 amino acids and a single stabilizing disulfide bond [69,70]. Of the three structural domains, DIII is the major antigenic domain of the E protein [62]. Mutation analysis revealed that it contains many potent NT epitopes and the primary receptor-binding motifs related to flavivirus [63]. For instance, the epitope EXE/DPPPFG is a cross-reactive epitope located in this region, and it is conserved among most flaviviruses, including ZIKV, WNV, YFV, and JEV. This region has received attention for its potential as a diagnostic marker or a target for vaccine and treatment development [71,72]. Nuclear magnetic resonance spectroscopy predicts that the location of those neutralizing epitopes on DIII is similar among different flaviviruses [62].

Several studies [44,73] have shed light on the significance of the E protein dimer’s quaternary epitopes as critical targets for cross-neutralizing antibodies across the Flaviviridae family. The conservation of these dimeric epitopes is attributed to the structural arrangement required for E protein function during viral fusion and maturation [74], so they represent promising targets for the development of a pan-flavivirus vaccine.

Using x-ray crystallography, Zhao and colleagues identified six mAbs against the DIII capable of neutralizing multiple strains of ZIKV in mice [75]. Other studies with bnAbs from human serum have reached similar conclusions, demonstrating that bnAbs provide protection not only to ZIKV, but also to DENV-1 [76]. These bnAbs primarily inhibited viral entry by locking the E dimer in a pre-fusion state, preventing the conformational rearrangement required for membrane fusion [77].

Together, available data suggest that the conserved quaternary epitopes on the E protein dimer constitute a structurally and functionally critical target for pan-flavivirus immunity. Continued structural and immunological characterization of these epitopes will be instrumental in guiding the rational design of flavivirus vaccines and therapeutics.

### 2.2. Role of E-Dimer and Higher-Order Structures in Viral Entry

**Figure 2:**
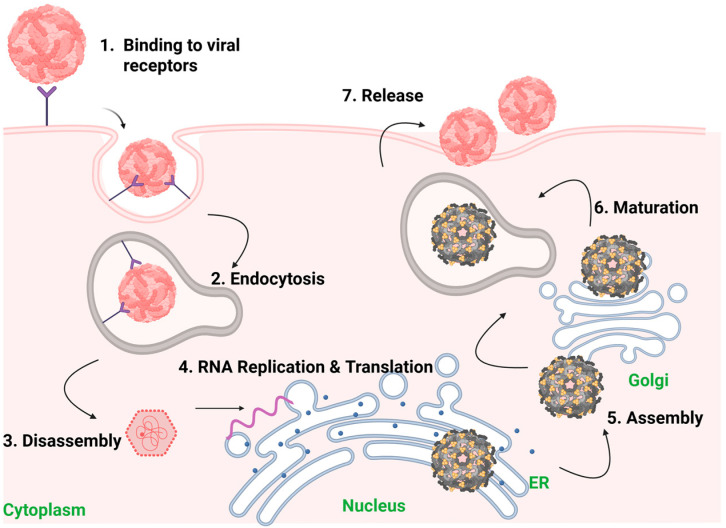
Flavivirus life cycle. Virus particles bind to specific receptors of the host cell (1) and are internalized by clathrin-mediated endocytosis (2) [78]. The acid environment of the endosomal vesicle induces conformational changes in DII, leading to the release of the viral genome into the cytosol (3). The RNA is translated into a polyprotein, originating seven non-structural and three structural proteins (4) (shown in Figure 1B) [79]. After that, the virion is assembled and matured for release on the surface of the ER and trans-Golgi (5). The host protease furin cleaves prM to M, producing mature infectious particles (6). Then the mature virion follows the secretion pathway and is released by exocytosis (7) [80]. Created in BioRender. Pinzon, E (2025) https://app.biorender.com/illustrations/69169f4c8fc82e5077c32b46.

Flaviviruses utilize sophisticated and highly coordinated mechanisms to enter host cells. Central to this process is the E protein. Upon endocytosis of the virus by the host cell, the mildly acidic environment of the endosome triggers a substantial rearrangement of the E dimers into fusogenic trimers. This low-pH-dependent transition exposes the hydrophobic fusion loop of the DII, which inserts into the endosomal membrane and facilitates membrane fusion between the viral envelope and the endosomal membrane [60] (Figure 2).

The DI-DII hinge is also involved in conformational changes of the E protein during virus entry. Fibriansah and colleagues showed that the highly neutralizing 5J7 antibody (which binds to the DI-DII hinge region in DENV3) can remove the virus from the cell surface [81]. This has also been shown in WNV with the CR4354 antibody [82] and in DENV1 with 1F4 [83].

The polymerization of E proteins is a tightly regulated process that is also critical for receptor binding and membrane fusion. Structural studies of DENV and ZIKV have demonstrated that the disruption of dimer formation or trimer assembly abrogates infectivity [61]. Modis and colleagues showed that the pre-fusion dimer configuration provides stability to the virion at neutral pH, while allowing for rapid activation of fusion machinery upon acidification [60]. Furthermore, cryo-electron microscopy studies revealed that temperature can modulate the conformational flexibility of E-dimers on the viral surface, particularly in ZIKV and DENV. This structural plasticity is thought to influence viral tropism and immune recognition [84,85]. Hence, the E-dimer represents a prefusion form of the flavivirus envelope protein, whose transition to higher-order oligomers allows membrane fusion, an essential step for viral entry [58,86].

### 2.3. Known Quaternary Epitopes Target the E Dimer

The accessibility of quaternary epitopes is influenced by the inherent conformational flexibility of the virion. Kinetic studies on WNV and DENV infection detail how the neutralizing potencies of the antibodies are determined by their affinity to the virion, the number of sites available for binding, and the conformational structure of the E protein. The E protein, under specific physical and chemical conditions, can expose certain epitopes that increase neutralization [87].

The implications of this observation for vaccine design rely on the fact that immunogens must faithfully represent the native quaternary structure of the E dimer to potentially elicit protective immune responses. Additionally, their conservation across multiple flaviviruses makes them key targets for cross-reactive neutralizing antibodies [87].

- E Dimer Epitope 1 (EDE1)

**Figure 3:**
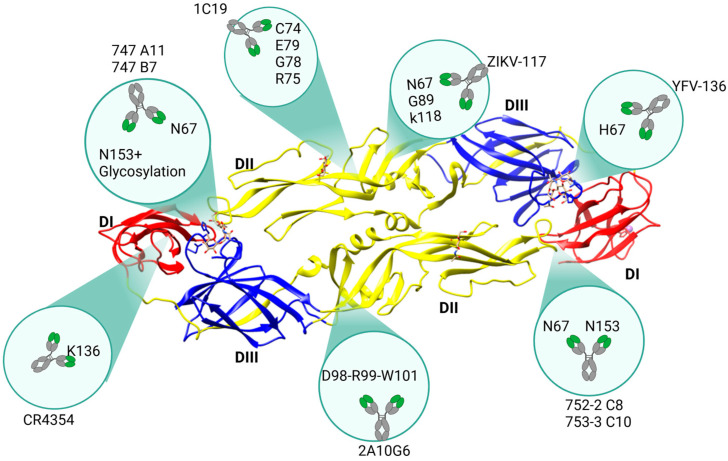
Structural model of E-dimer, highlighting the immune complex between antibodies and conserved quaternary epitopes. Antibodies are shown in grey. DI, DII, and DIII are shown in red, yellow, and blue, respectively. Inside the circle, the amino acid residues key to the proper binding of the specific antibody are shown. Neutralization values of these antibodies are depicted in Table 1. The DENV-2 Envelope Protein structure (PDB ID:10AN) was obtained from the RCSB Protein Data Bank [60] and visualized with UCSF Chimera, developed by the Resource for Biocomputing, Visualization, and Informatics at the University of California, San Francisco, with support from NIH P41-GM103311. Created in BioRender. Pinzon, E (2025) https://app.biorender.com/illustrations/694c38e472755df4b1e8724b.

First described in 2015 by Rouvinski and colleagues, 752-2 C8 and 753 C10 are potent bnAbs that target two highly conserved N-linked glycosylation sites at positions N67 and N153 of the DI/DII interface (Figure 3) [88]. This binding prevents the E dimer rearrangement and fusion loop exposure, potently neutralizing DENV serotypes 1, 2, and 3, and weakly inhibiting DENV-4 (Table 1) [89]. This cluster of epitopes is conserved not only across the four serotypes of DENV but also in ZIKV, as shown by Barba and colleagues [47].

b. E Dimer Epitope 2 (EDE2) Dimer Epitope 2

747 A11 and 747 B7 are two antibodies against the interface of the DI/DII proteins, recognizing glycans at N-67 and N-153 as EDE1, but requiring additional glycosylation at position N153 for efficient binding (Figure 3). Their neutralization mechanism is thought to involve blocking the low-pH-induced rearrangement of the E protein. Unlike antibodies against EDE1, the quaternary epitopes recognized by EDE2 bnAbs are highly conserved among DENV serotypes, albeit less conserved in other flaviviruses [88].

c. Zika Virus Envelope Dimer Epitope (ZVEDE)

Cryo-electron microscopy showed that the ZIKV-117 antibody cross-links the monomers within DII (Figure 3), preventing the reorganization of the E protein monomers into fusogenic trimers within the acidic environment of endosomes [77]. Although these quaternary epitopes are highly conserved among different strains of ZIKV (Table 1), they are not as conserved on the other flaviviruses. Therefore, ZIKV-117 does not neutralize any of the DENV serotypes or WNV [90]. Nevertheless, the antibody can markedly reduce tissue pathology, placental and fetal infection, and mortality in ZIKV-infected mouse models [90].

d. WNV Lateral Ridge

CR4354 is a human WNV-E-specific antibody that potently neutralizes WNV and, less potently, DENV and other flaviviruses. Although K136 is the key residue in DI recognized by the antibody, 18 additional consensus residues are identified at the contact interface, including amino acids present in DII and DIII. Little to no conservation of the identified epitope residues was observed among other flaviviruses, explaining the strict specificity of CR4354 for WNV (Figure 3) [82]. A conformational locking of the E protein appears to be the mechanism of neutralization. The discovery of this human antibody is important because prior WNV candidate vaccines have only been tested in mice [91], hamsters [92], and horse models [93]. There is no good correlation between in vivo protection or severe disease in humans and animal models, mainly because animal models generate different antibody responses against common specific WNV epitopes. Thus, understanding the molecular path of action of this human antibody could greatly improve current knowledge of the in vivo protection from WNV infection in humans [94].

e. YFV Dimer Epitope

Antibody YFV-136 was isolated from a panel of YFV-specific human mAbs targeting the dimeric interface of the FL and adjacent DI residues (Figure 3). It exerts broadly neutralizing activity by locking the dimer in a prefusion state [95]. It has a high neutralization potency (IC_50_ of <10 ng/mL), making it one of the most potent mAbs against YFV to date [96]. Of note, this antibody binds to monomeric YFV E protein. Efforts are underway to identify antibodies targeting quaternary structural epitopes, including dimeric forms, against YFV E protein, as seen for many flaviviruses. Yet, ongoing efforts have not identified neutralizing mAbs thus far [97].

f. Cross-Flavivirus Fusion Loop Proximal Epitope

2A10G6 is a potent antibody against the DRXW motif in the highly conserved N-terminal fusion loop at the tip of DII (Figure 3). Crystal structure of the DENV 2 E protein revealed three amino acids (D98, R99, and W101) exposed on the virion surface and constituting a quaternary epitope highly conserved among flaviviruses, including DENV 1-4, WNV, JEF, and YFV. In a BALB/C mouse model, 2A10G6 neutralized DENV 1-4, YFV, and WNV, and protected from lethal challenge with DENV 1-4 and WNV [98].

In addition, 2A10G6 neutralized and protected against ZIKV in an A129 mouse model. Currently, efforts are underway to humanize 2A10G6 for the treatment of flavivirus infection in humans [99].

1C19 is another recently developed antibody that effectively neutralizes and reduces viremia in DENV- and ZIKV-challenged AG129 mice. 1C19 targets a novel epitope, RCPTQGE, located on the amino acids 73 to 79 of EDII (Figure 3). Serum from animals infused with 1C19 and challenged with either DENV or ZIKV was negative for ADE, both in vitro and in vivo ADE assays. These findings highlight the potential of this epitope region as a promising target for pan-flavivirus vaccine development [100].

The potential of these quaternary epitopes to become effective targets for a pan-flavivirus vaccine is influenced not only by the paratope/epitope complementarity but also by the topological distribution at the immunogen’s surface and, most importantly, the similarity to the mature viral particle. Continued structural and immunological characterization of these epitopes will be instrumental in guiding the design of a successful pan-flavivirus vaccine [101].

**Table 1:** Summary of known antibodies targeting quaternary epitopes in the E dimer of different flaviviruses.

Antibodies	Flavivirus	Epitope	Neutralization Values/ Technique	Reference
Z004	ZIKV	EDIII	0.7 ng/mL FRNT	[76]
	DENV1		1.6 ng/mL FRNT	
752-2-C8	DENV1		0.39 nM FRNT	[47]
	DENV2 DENV3 DENV4 ZIKV	EDE1	0.24 nM FRNT 0.64 nM FRNT 1.13 nM FRNT 0.095 nM FRNT	
753(3)-C10	DENV1		0.54 nM FRNT	
	DENV2 DENV3 DENV4 ZIKV	EDE1	0.18 nM FRNT 1.89 nM FRNT 0.08 nM FRNT 0.063 nM FRNT	[47]
747(4)-A11	DENV1		0.11 nM FRNT	
	DENV2 DENV3 DENV4 ZIKV	EDE2	0.07 nM FRNT 0.11 nM FRNT 7.79 nM FRNT 0.904 nM FRNT	[47]
747(4)-B7	DENV1 DENV2 DENV3 DENV4 ZIKV	EDE2	0.10 nM FRNT 0.11 nM FRNT 0.12 nM FRNT 93.19 nM FRNT 4.31 nM FRNT	[47]
ZIKV-117	ZIKV	EDII	5.4 ng/mL IC_50_	[77]
CR4354	WNV	EDI-II-III	26 ng/mL PRNT	[91]
YFV-136	YFV	EDII	5.5–9.2 ng/mL FRNT	[96]
2A10G6	DENV1 DENV2 DENV3 DENV4 YFV WNV ZIKV	EDII	2 µg/mL PRNT 1.5 µg/mL PRNT 2.1 µg/mL PRNT 1.8 µg/mL PRNT 3.6 µg/mL PRNT 46 µg/mL PRNT 249 µg/mL PRNT	[98,99]

FRNT: focus reduction neutralization test. IC: Half maximal inhibitory concentration. PRNT: plaque reduction neutralization test.

## 3. Immunogenic Landscape of Immune Responses to Flavivirus

Flaviviruses elicit complex and dynamic immune responses involving both the innate and adaptive arms of the immune system. Upon infection, the innate immune system detects viral RNA through pattern recognition receptors (PRRs) such as RIG-I and MDA5. These PRRs trigger type I interferon (IFN) production and inflammatory cytokine release to limit viral replication [102,103]. Adaptive immunity plays a key role in viral clearance and long-term protection. In the adaptive response, B cells produce virus-specific antibodies, which can neutralize viral particles [104]. T cell responses, particularly CD8+ cytotoxic T lymphocytes, are also essential for controlling infection. However, for both B and T cells, cross-reactivity among flaviviruses can result in either protective or pathogenic outcomes, depending on epitope specificity and host genetics [105].

### 3.1. Virus-Specific Tropism and Pathogenesis

DENV. In DENV infection, monocytes are the main cellular target. However, B cells represent another possible target for the virus. Lymphoid organs are targets of DENV infection, with viral antigens detected inside blast cells of B cell follicles, plasma cells, and B cells in the spleen and lymph nodes [106]. Additionally, active viral replication of DENV in the human germinal center has been confirmed by the detection of positive-strand DENV RNA in the spleen [107]. At the peripheral blood level, in a pediatric population of patients with severe Dengue, viral RNA was detected in 40% of B cells, but only 5% of monocytes and NK cells [108]. However, B cells showed low expression of protein E and fewer virions compared to monocytes.

The mechanisms of B-cell activation, maturation, and differentiation during dengue infections are important in the clinical outcome of the disease. The specific role of plasma blasts and plasma cells during DENV infection has been evaluated for years [109]. The acute phase of DENV infections, especially in pediatric patients with severe dengue, is characterized by a shift of the B-cell subset toward plasma blasts and plasma cells (PCs) [110]. One study comparing patients with severe and mild dengue found a 5.4% and 7.2% increase in plasma blasts (CD27hi CD38hi CD138’−) and plasma cells (CD27+ CD38hi CD138+), respectively; and a 15% decrease in naïve B cells (CD27−) and a 2-fold increase in IL-10-producing B cells, with regulatory functions (Breg-CD27+ CD38hi CD138+) [111].

The population of plasma blasts in adult patients reinfected with DENV of a new serotype showed that memory-derived plasma blasts represent 51–80% of all peripheral B lymphocytes (as a reference, this population represents 1–5% in healthy adults). In these adult patients, 60–70% of isolated plasma blasts secreted dengue-specific, but cross-reactive serotype-reactive, IgG antibodies in vitro (binding specificity of 81% to the E protein) [112].

Characterizing IgG antibody profiles in DENV is essential for guiding vaccine design and predicting clinical outcomes. Understanding this repertoire helps elucidate how specific IgG responses can mediate protection or promote ADE.

DENV-antibody complexes trigger inhibitory cascades in FcγRI/IIA-bearing THP-1 cells, including DAK, Atg5-Atg12, SARM, and TANK, which impair RIG-I/MDA5 and TLR3/4/7 signaling. As a result, there is reduced production of type I interferons and diminished expression of IL-12, IFN-γ, TNF-α, and nitric oxide (via iNOS suppression) [113]. Concurrently, ADE promotes IL-10 production, which further suppresses antiviral responses through SOCS-mediated inhibition of JAK/STAT signaling, favoring a Th2-biased, anti-inflammatory environment. Silencing IL-10 abrogates these effects, highlighting its central role [114]. Collectively, ADE not only enhances viral entry and induces excessive cytokine signaling and vascular leakage but also reprograms host innate and adaptive immune responses to facilitate increased DENV replication.

JEV. Most of the infections are primarily asymptomatic or mild, but about 1% of those infected progress to neuroinvasive disease. Among serious cases, ~30% recover, while another 30–50% develop permanent neurological sequelae, and ~25% die [115]. Complications of the disease are encephalitis or meningoencephalitis, with seizures, paralysis, altered consciousness, and movement disorders, often linked to poor outcomes due to elevated intracranial pressure. Histology of JEV-infected brains reveals perivascular central nervous system (CNS) inflammation and neuronal infection, particularly in the thalamus and brainstem, leading to long-term neurological deficits or death [116].

JEV initially replicates in peripheral immune cells, such as fibroblasts or macrophages, and in local lymph nodes, which typically control the infection and prevent progression to CNS disease. In the periphery, JEV replicates preferentially in Ly6Chi CCR2^+^ macrophages/dendritic cells derived from monocyte precursors, which act as viral reservoirs with the ability to infiltrate the CNS and amplify neuroinflammatory responses [117].

The BBB can be disrupted by JEV-infected mast cells via the release of proteases [118]. It can also be disrupted by JEV-infected astrocytes, which secrete IL-6, VEGF, MMP-2, and MMP-9, leading to ubiquitin-proteasome-mediated degradation of tight junction proteins (ZO-1, claudin-5) in endothelial cells. This process, amplified by proinflammatory cytokines from activated microglia, disrupts the BBB through JAK2/STAT3 signaling and induction of n-recognin-1 [119,120].

Upon CNS invasion, JEV activates microglia (brain resident macrophages) and astrocytes, triggering multiple innate immune signaling pathways, including cascades mediated by TLR3, RIG-I, and ROS [121]. In turn, these pathways drive the production of TNF-α, IL-6, CCL5, CXCL10, IFN-γ, and CCL2, which mediate neuroinflammation and associated neuronal damage [122]. In microglia, NLRP3 inflammasome activation, caspase-1 cleavage, and CCR2 upregulation further promote neurotoxic phenotypes [123,124]. Activated microglia also secrete extracellular vesicles containing let-7a/b, which induce caspase-mediated neuronal cell death and highlight the important interaction between glial and neuronal cells [125].

Furthermore, JEV sub-genomic RNA interacts with TLR7 to suppress type I IFN responses, while TLR7 deficiency induces compensatory signaling mediated by TLR8. This highlights the complex interaction of TLRs in modulating CNS immune responses during JE pathogenesis [126,127].

T cell-mediated immunity is critical for protection against JEV. CD4+ T cells promote B cell activation, antiviral cytokine production (e.g., IFN-γ), and memory formation. CD8+ T cells contribute primarily to asymptomatic individuals by viral clearance during the late phases of infection. Protection against severe viral diseases, including encephalitis, is primarily mediated by humoral immunity. Neutralizing IgG antibodies against the viral envelope protein (E) are crucial for this protection. Antibodies against NS1 and prM play different roles; anti-NS1 antibodies act through non-neutralizing mechanisms, while anti-prM antibodies can actually enhance disease [128,129]. Myeloid-derived suppressor cells (MDSCs) can affect CD4+ T cell responses, reducing splenic B cells (CD19+) and plasma cell (CD19+CD138+) populations and lowering IgM and neutralizing antibody levels [130].

WNV. Most infections (80%) are asymptomatic, while symptomatic cases range from fever and flu-like illness to severe neurological disorders. Severe cases occur in 1 in 150 infected individuals, and among these, 50–71% develop encephalitis, 15–35% develop meningitis, and 3–19% develop acute flaccid paralysis. Of the severe cases of encephalitis, between 3% and 19% are fatal, while survivors usually recover physically and mentally within a year [131,132].

In the initial phase following subcutaneous infection, WNV initially targets keratinocytes, dermal dendritic cells, and Langerhans cells. Infection in keratinocytes triggers innate cytokine responses via TLR-7, promoting migration of Langerhans cells to draining lymph nodes [133,134]. Here, the virus amplifies, resulting in viremia and spreading to organs such as the spleen, kidneys, and other visceral tissues. Likely targets in these tissues include subsets of dendritic cells, macrophages, and neutrophils. Following systemic infection, WNV can cross the BBB and invade the CNS. Neuroinvasion is influenced by the virus’s proteins, particularly by an N-linked glycan on the E protein, which can enhance binding and penetration of endothelial cells [135]. The virus can enter through the alteration of the BBB, regulated by vasoactive cytokines (TNF or other inflammatory mediators such as MIF and ICAM1 induced by TLR3). WNV can also enter the brain by increasing the permeability of the BBB through MMP9 (degradation of tight junction proteins) [136,137]. The virus crosses the BBB by transcellular or paracellular direct infection of endothelial cells, or by retrograde axonal transport through peripheral motor nerves [138].

The neuropathogenesis of WNV depends on the virus’s ability to enter the CNS and replicate in target cells such as neurons, astrocytes, and microglial cells. These resident cells of the CNS can generate strong innate immune responses against WNV without systemic immunity. Their susceptibility varies by cell type, depending on intrinsic defense programs. ISG-Ifit2 (interferon-stimulated gene) limits viral spread [139], and cerebellar granular neurons, with strong STAT1- and IFN-dependent pathways, are more resistant than cortical neurons [140]. However, in these cells, type I IFN production does not depend on TLR3 as it does in DCs or macrophages [141]. Multiple PRR pathways, including NLRP3-inflammasome, MyD88, and TLR3, help restrict replication and are required for protection against lethal WNV infection [142,143].

Caspase-12 also regulates IFN antiviral responses via TRIM25 and RIG-I [144]. In microglia, WNV induces TNF-α, IL-6, and IFN-β via TLR3 [145], but it has been associated with neuronal death, given the pro-inflammatory M1 polarization. Studies suggest that M1 microglia are required for control of WNV infection, and the M2 phenotype (anti-inflammatory) may prevent neuronal death and tissue damage. Astrocytes reduce susceptibility by inhibiting protease activity [146]. In addition, WNV infection triggers the release of chemokines, facilitating migration of immune cells across the BBB, such as CXCR3+ CD8+ T cells migrating to the cerebellum, guided by CXCL10, through the activation of microglia [147].

### 3.2. Implications for Vaccine Design

The immunological landscape of flavivirus infections provides an informed background for designing safe and effective vaccines that balances humoral and cellular immunity and minimizes the risk of ADE, which is a main concern following Zika or Dengue infection because of the production of cross-reactive antibodies directed against DI/II. In vitro and in vivo, these antibodies offer little neutralization and instead potently drive ADE, increasing the risk of severe disease from subsequent infection with a different flavivirus [29].

## 4. Vaccine Evaluation and Translational Insights

### 4.1. Preclinical Insights

Recent work in mouse models highlights the superiority of stabilized E-dimer immunogens in promoting robust neutralizing IgG responses. Using BALB/c mice, Campos and colleagues showed that mice immunized with an engineered covalent E-dimer achieved significantly higher neutralization titers compared to monomeric E immunized mice [148]. The neutralization assays against ZIKV and DENV were performed using a focus reduction neutralization test (FRNT). The FRNT_50_ for E monomer and E-dimers were 496.4 and 535.8, respectively. The mice challenged with the E dimer were less prone to ADE compared to those injected with the monomeric version [148]. In the same study, using C57BL/6 wild-type mice to assess the efficacy of the E-protein vaccines against ZIKV in pregnancy, the E-dimer conferred better protection than the monomeric E, with substantial reductions in the burden of ZIKV RNA in fetal specimens. This underscores how E-dimer stabilization conceals undesirable FL epitopes (prone to ADE) while presenting protective, dimer-dependent epitopes more effectively [148].

Recently, VLP technology has been used to engineer a novel vaccine candidate against DENV and ZIKV. It is based on genetically fusing the DIII domain of ZIKV and DENV to the AP205 dimer, using VLPs as a vaccine platform. In C57BL/6 mice, the vaccine elicited strong DIII-specific IgG responses; when these antibodies were tested in vitro, no significant ADE was observed. The team is conducting a murine challenge testing the protective efficacy [149].

### 4.2. Clinical Insights

In human trials, important milestones have been reached in species-specific flavivirus vaccine development, particularly for DENV and ZIKV. Phase I randomized, placebo-controlled trials in the U.S. and Puerto Rico compared TV003 and TV005 vaccines (developed by NIH) against DENV. The vaccines demonstrated good safety profiles in 18–50-year-old participants, with seroconversion rates of up to 100% in flavivirus-experienced participants. Ongoing Phase II and III trials are currently ongoing in Taiwan and Brazil [150].

TAK-003 (also known as DENVax or Qdenga), a live-attenuated recombinant vaccine targeting DENV 1-4, has undergone Phase I and II trials in multiple countries. A Phase III trial, including 20,099 participants across eight dengue-endemic countries, was completed recently. The trial demonstrated a 61% efficacy against virologically confirmed dengue, and 54% efficacy against confirmed dengue in seronegative individuals over 4.5 years of age [10].

Results from several ZIKV vaccine candidates have been published recently. A single dose of ZIKV purified inactivated vaccine (ZPIV) in a DENV-experienced human elicited potent cross-neutralization antibodies to both ZIKV and DENV. Notably, one mAb termed MZ4, which targets a linker region on the EDI-EDII region, protected BALB/c and C57BL/6 mice from viremia and viral dissemination following ZIKV or DENV-2 challenge, respectively. Additionally, ZPIV vaccination in Puerto Rican individuals with prior flavivirus experience reported a similar response [151].

Notably, leading mRNA and DNA platforms in early-phase human trials are demonstrating strong immunogenicity in humans, mice, and non-human primates. Two DNA vaccines developed by the NIAID’s Vaccine Research Center, VRC5283 and VRC5288, have shown great T cell responses at four weeks after a needle-free administration [27].

mRNA-1893 is a Moderna vaccine candidate that has completed a Phase 1 trial [152]. Results were presented as geometric mean titers from the 50% plaque reduction neutralization test (PRNT50 GMTs), the gold-standard method for assessing the immune response to viral infections. At the 100 µg vaccine dose, PRNT50 GMTs were 45.9 in flavivirus-negative participants and 130.6 in flavivirus-positive participants [153]. The phase II trial (NCT04917861) has been completed, but the quality-control review is still pending.

Finally, TAK-426, Takeda’s purified inactivated ZIKV vaccine, completed a phase I clinical trial with an acceptable safety profile and was immunogenic in both flavivirus-naïve and flavivirus-primed adults [26]. Seropositivity for neutralizing antibodies was 100% at 1 year in both groups. Seropositivity in the 2 years was 93.8% and 76.2% in the FV-naive and FV-primed groups, respectively. Based on the safety and immunogenicity profiles, the 10 ug TAK-426 dose was selected for further clinical development [154].

### 4.3. Limitations and Variability in Responses

- Animal Model Limitations

Flavivirus research has been hampered by the lack of animal models that accurately replicate human disease. For example, DENV does not naturally infect mice because DENV proteins (e.g., NS5) are highly host species specific. Therefore, recapitulation of significant dengue disease requires intracerebral inoculation or the use of immunodeficient animal models [155]. Even when more appropriate mouse models are used, such as virus-adapted models, differences in the variable regions of the heavy and lambda light chains of the antibodies produced [156], as well as genetic differences in major histocompatibility complex (MHC) class I and II molecules [157], prevent extrapolation of the animal findings to humans [158].

ADE is especially challenging to study in mouse models because in vitro ADE assays might score differently from one assay to another. This is due in part to the presence or absence of the Fcγ receptor on the target cells, differences in expression of viral receptors, and differences in cytokine responses [159].

Non-human primates, on the other hand, can be infected with DENV and maintain low viremia. However, key signs of viral human infection, such as febrile episodes, are missing [160,161]. Moreover, monkey models are often constrained by ethical, logistical, and financial limitations that restrict the number of animals used per study, decreasing the statistical power of outcomes [160,162].

In line with this, many of the clinical features of other human flavivirus infections are not recapitulated in commonly used animal models. For example, neurological manifestations of ZIKV, such as Guillain-Barré syndrome or microcephaly, are difficult to mimic in adult animals. Neurological manifestations can be observed by intravenous inoculation of high ZIKV titers, a route that bypasses the innate immune response within peripheral organs that normally occurs in natural human infection [163].

Ongoing efforts to develop more physiologically relevant models, including humanized mice [164] and mouse-adapted flaviviruses for the development of efficient animal models [165], could help bridge these translational gaps.

b. In Vitro vs. In Vivo Neutralization Correlation

There is a lack of correlation between the titers of protective antibodies and the disease severity or peak viremia for DENV in secondary infections [166]. Laoprasopwattana and colleagues conducted a prospective cohort study in 148 schoolchildren who received a diagnosis of acute symptomatic secondary DENV infection. The study measured enhancing activity, levels of NAbs, and viremia. There was no correlation between the neutralizing capacity at the time of infection and the disease severity [166]. This lack of correlation for DENV has also been observed in efficacy clinical trials. To note, some DENV studies estimate the efficacy of Dengvaxia as low as 70% after three injections [167].

Concerns have been raised about the accuracy of PRNT_50_ to assess real protection, highlighting the complexity of correlations of protection for flaviviruses. Moreover, although PRNT is the gold standard, it often fails to predict actual protective immunity against flavivirus, especially in the context of cross-reactive antibodies [168]. The origin of the DENV strain is also a factor to note in PRNT assays. Studies suggest that viruses produced in tissue culture are less mature than those produced in primary cells. This lack of maturation has a direct effect on the arrangement of the dimers on the surface of the protein E, which alters antibody recognition and neutralization, as previously noted [169]. To address this, Mukherjee and colleagues developed a stable Vero Cell Line that expresses high levels of human furin, allowing the production of homogeneous and mature flavivirus populations. However, it has not yet been standardized for use as a gold standard in neutralization and structural studies on flaviviruses [169].

c. ADE and Epitope Targeting

The development of an effective flavivirus vaccine is uniquely challenging due to the complex interplay of ADE. Sridhar and colleagues observed this effect while developing an anti-DENV NS1 ELISA in samples from three efficacy trials that tested the efficacy of the Dengvaxia vaccine. They concluded that Dengvaxia increased the risk of hospitalization for severe dengue among seronegative participants 2–16 years old, suggesting that, in the absence of previous DENV exposure, the vaccine increases the risk of severe dengue during subsequent infection. Interestingly, for participants who did have previous dengue exposure, Dengvaxia decreased the risk of severe dengue, which highlights the complex challenges that ADE poses to vaccine development [170].

The mechanism of ADE involves the production of cross-reactive, sub-neutralizing antibodies. These antibodies usually target conserved but non-protective epitopes, such as the fusion loop of EDII and the prM protein. As mentioned, these regions are often immunodominant, but they do not necessarily induce protective immunity and are strongly associated with ADE [28]. In contrast, antibodies targeting DIII are typically more type-specific and neutralizing, yet they tend to be underrepresented in the immune response following natural infection or vaccination [28,88].

Immunodominance can vary widely and skew responses toward epitopes that may not offer optimal protection. Some vaccines tend to elicit a broad but unbalanced immune response. As an example, a vaccine trial assessing the efficacy of TAK-003 demonstrated 97.7% efficacy against DENV2, 73.7% efficacy against DENV1, 62.6% efficacy against DENV3, and inconclusive results for efficacy against DENV4. These results raise concern about incomplete protection and possible ADE in future heterologous exposures [171].

The problem of variable epitope is further compounded by individual host factors such as HLA. In Southern Brazil, for instance, the HLA-DRB1*15 and DQB1*06:11 alleles have been reported as a susceptibility type in DENV3 [172]. Later, Weiskopf and colleagues reported HLA-DRB1 alleles associated with different magnitudes of DENV-specific CD4+ T cell responses. Alleles DRB1*0301, DRB1*0403, DRB1*0802, DRB1*1101, and DRB1*1502 were linked to responses of lower magnitude, potentially influencing disease severity. However, interactions with CD8+ T cells or antibody responses may also influence the immunological response seen in certain flavivirus infections [173]. Furthermore, HLA-B∗44 supertype alleles have also been associated with increased susceptibility to severe dengue in a cohort of DENV patients from three hospitals in Recife, Brazil [174]. These advances toward understanding the effect of HLA on flavivirus infections have provided new strategies for improving the design of dengue vaccines [175]. For example, Roth and colleagues successfully tested immunogenicity and protection of an mRNA vaccine in human HLA class I transgenic mice, targeting immunodominant T cell epitopes that generated potent virus-specific T cell responses. This mRNA vaccine conferred immunity in a mouse model of DENV infection [176].

Among these newer vaccine strategies designed to overcome the limitations posed by HLA allele heterogeneity, epitope engineering and T cell-oriented designs are emerging as potential candidates. Along with Campo and colleagues’ work, discussed earlier [148], several other studies have shown that point mutations in the fusion loop and adjacent residues can reduce the generation of ADE-prone antibodies without significantly compromising immunogenicity [177,178].

As an example, E106 is a DNA vaccine that uses a template of the JEV E protein with a mutation in the 106th amino acid residue. This region was found to promote the production of ADE-prone monoclonal antibodies cross-reactive to DENV and JEV. The version of the vaccine with the mutation protected BALB/c mice from JEV infection while reducing ADE-prone cross-reactive antibodies by 64-fold compared to the wild-type JEV DNA vaccine [179].

## 5. Design Strategies for Stabilizing Quaternary Epitopes

A growing body of evidence has established that protective neutralizing antibodies tend to recognize quaternary epitopes on the surface of flaviviruses (Figure 3). These epitopes are formed only when the viral E protein assembles into dimers or trimers. These conformational changes triggered by the virion’s exposure to low pH, were predicted by Kuhn and colleagues [180]. Using cryogenic electron microscopy, this research team created a three-dimensional image reconstruction of DENV showing the various E protein conformations. Accordingly, mature viruses undergo large rotations that allow the arrangement of the protein units in their surface as dimers (which further assemble into a smooth herringbone lattice of 90 dimers). Conformational changes for dimerization also shift the fusion loop into an inaccessible region while also exposing surface area [180]. The new surface area appears to have a greater-than-average membrane curvature, resulting in smaller vesicles, as seen in electron micrographs reported by Modis and colleagues [181]. Furthermore, this property may help promote the fusion of viral and host–cell membranes [181].

These quaternary epitopes are structurally different and poorly mimicked by monomeric antigens. Exposing elements, such as the fusion loop, elicits poor neutralizing antibodies [182,183]. When used in subunit vaccine designs, monomeric versions of the E protein often induced poorly neutralizing or cross-reactive antibodies, which might exacerbate ADE rather than confer protection [184,185].

The antibodies that target quaternary epitopes have been recently investigated to explain the broad cross-protection observed in dengue patients with multiple prior DENV exposures. Recently, Mpingabo and colleagues reported that antibodies targeting the quaternary epitope, called the envelope dimer epitope (EDE), were associated with broad neutralization of mature DENV1-4 viruses in a cohort of 2996 participants in the Philippines [186]. The broadly neutralizing character of these antibodies strongly suggests that broad-spectrum vaccines and therapeutics for DENV are possible [186].

Recent vaccine development efforts have focused on strategies that recapture this phenomenon by stabilizing and presenting native-like quaternary epitopes through recombinant VLPs, and mRNA-encoded, multivalent antigen platforms to develop safer and more effective vaccines against flaviviruses [187].

### 5.1. Protein Engineering Approaches

Protein engineering strategies, such as the implantation of disulfide bridges, have been employed to stabilize the flavivirus-E protein dimer conformation [70]. This strategy was successfully applied by Rouvinski and colleagues to lock the E dimer in a native-like conformation and reduce the exposure of the immunodominant fusion loops. The strategy promoted recognition of conserved quaternary epitopes such as EDE [183].

Zhu and colleagues developed a chimeric, recombinant virus encoding DENV2 DI, DII, and DIII sequences in the DENV4 E glycoprotein backbone, which displayed a high density of quaternary epitopes [188]. This construct immunized BALB/c mice against DENV, eliciting higher levels of DENV2-neutralizing antibodies compared to mice vaccinated with the monomeric form of the protein. This study provided proof-of-concept for leveraging structure-based design to produce vaccines for dengue and other flaviviruses using quaternary epitopes [189].

The high abundance of monomers may help explain the poor performance of DENV subunit vaccines observed to date. Thiono and colleagues recently investigated the specificity and epitope targets of the NAbs elicited by either monomer or dimer versions of the E protein antigens. They found that the responses were more effective when the antigens closely resembled the viral surface and presented conserved quaternary epitopes, such as those presented in the dimer version [190].

Protein engineering techniques are also being used to test how single mutations in the E protein could hamper the induction of ADE-prone antibodies. Weiß and colleagues immunized BALB/c mice with a recombinant vaccine bearing a mutated version of the E protein (specifically the FL region) against WNV infection. They showed a significant decrease in the ADE activity against ZIKV in WNV-vaccinated animals [42].

Together, these approaches demonstrate that structural engineering of the E protein has the potential to develop vaccine subunits for the E protein’s protective, quaternary epitopes.

### 5.2. Virus-like Particles (VLPs) and Nanoparticle Platforms

Another powerful strategy involves the use of VLPs, which mimic the architecture of the native virion but lack infectious viral RNA. Moreover, the particulate form of VLPs makes them more immunogenic than subunit vaccines, a clear advantage when using limited antigenic sites as targets [50]. VLPs have been successfully used since the first recombinant human vaccine against the hepatitis B virus in 1986 [191]. Subsequently, iterations include vaccines against the human papillomavirus [192], SARS-CoV-2 [193], and Respiratory Syncytial Virus [194].

VLP-based vaccines have been widely explored in flavivirus research. Côrtes and colleagues developed a self-adjuvanted VLPs-based vaccine displaying the ZIKV DIII, which elicited a strong Th1-biased immune response and protected C57BL/6 mice from ZIKV-induced cerebral and testicular damage [195].

In fact, VLPs are particularly well-suited for flavivirus vaccine development because the co-expression of prM and E proteins in mammalian, insect, or plant expression systems leads to the spontaneous assembly of particles displaying native E-dimer lattices [196,197]. These particles preserve the geometric constraints necessary to expose EDE and other quaternary epitopes while minimizing immunodominant non-neutralizing regions. Similarly, Yang and colleagues engineered a plant-produced VLP vaccine based on the hepatitis B core antigen that displayed ZIKV DIII epitopes, while deliberately excluding the fusion loop and adjacent domain II regions. This vaccine protected C57BL/6 mice against multiple strains of ZIKV without inducing the ADE effect or eliciting non-neutralizing antibodies against other flaviviruses [198].

Structural and immunological studies have demonstrated that the most potent and broadly neutralizing antibodies elicited during natural flavivirus infection preferentially recognize such quaternary E-protein interfaces [88,199].

A recent study reported a tetravalent VLP vaccine incorporating an F108A mutation in the E protein, which improved particle stability and immunogenicity. Immunization protected all non-human primates for up to one year against all four DENV serotypes, with no detectable in vitro ADE activity. This study demonstrated that a VLP-type vaccine is a promising candidate for inducing balanced, robust, and lasting immunity against not only DENV but also other flaviviruses such as ZIKV or WNV, due to similarity across this flavivirus-conserved structure [200]. This work was built on recombinant DENV1-4 VLPs previously developed by Urakami and colleagues, which carry a mutated fusion loop and co-express both pr M and E. These constructs induced high levels of neutralizing antibodies in vivo without any ADE activity in BALB/c mice and enhanced the VLP production for all four serotypes of DENV and ZIKV, by inhibiting E-protein-mediated cell fusion during VLP assembly [201].

Accordingly, mutations of the fusion loop region could help overcome one of the most challenging problems for flavivirus VLP production, namely, the formation of multinucleated cells. As shown by Charoensri and colleagues, the reduction in cell fusion could be achieved by mutating a hydrophobic residue in the fusion loop region [202]. However, this highly conserved region presents a potential target for the development of a pan-flavivirus vaccine. Thus, uncontrolled alterations could compromise the potential utility of a vaccine candidate targeting this region [202]. Studies are needed to understand how these modifications alter the neutralizing antibody and cell-mediated response in humans.

Similarly, Boigard and colleagues demonstrated that the conformation of the E protein displayed on the VLPs vaccines plays a critical role in the induction of highly neutralizing antibodies. BALB/c mice inoculated with VLPs displaying the protein E as a single polypeptide together with other structural viral proteins exhibited lower neutralizing titers when immunized with the version of the vaccine that was produced at 37 °C instead of 31 °C (a phenomenon known as molecular breathing [203]).

Molecular breathing of the E protein was described earlier by Fibriansah [81]. Zhan and colleagues showed how the shape of the mature virus changes when produced at 33 °C or 37 °C [204]. This is due to the effect that production temperature has on the folding and conformation of the E protein [203]. This study concluded that the protein conformation in the VLP vaccines could dictate the neutralizing antibody response. Moreover, it provided new information on how a balanced and robust immune response to flavivirus may require both the optimal quaternary epitopes and favorable environmental conditions during VLP production [203].

The first steps toward a pan-flavivirus VLP-based vaccine have already been taken. Recently, a mixed strategy consisting of designing VLPs with selected quaternary epitopes from EDE, using the C8 antibody (referenced earlier in this paper), was developed by Rouvinski and colleagues [88]. Immunization of BALB/c mice induced antibodies recognizing both ZIKV and DENV [205]. Currently, the team is evaluating the neutralizing activity of the antibodies and the effect on ADE burden. This represents a milestone in the use of quaternary epitopes as a target for a pan-flavivirus vaccine, which could lead to new approaches to designing more effective vaccines.

Emerging research highlights the importance of broadly neutralizing epitope mapping and B-cell repertoire characterization in rational flavivirus VLP vaccine design. Recent cryo-electron microscopy and structural studies have identified conserved quaternary epitopes on the flavivirus envelope protein that are shared across multiple species, offering promising targets for immunogenic engineering [206,207]. Additionally, longitudinal analyses of memory B-cell responses in flavivirus-exposed individuals show that lineage-targeted immunogens focusing on these quaternary surfaces can promote the maturation of broadly cross-neutralizing antibodies. These insights provide a strong conceptual basis for the development of pan-flavivirus VLP vaccines designed to elicit broad and durable immunity [208].

Understanding the human memory B-cell repertoire and its specificity for conserved flavivirus envelope epitopes is critical for the rational design of VLP-based vaccines that elicit broad protection. Analyses of flavivirus-infected individuals have shown that memory B-cell responses are shaped by clonal expansion and affinity maturation toward envelope protein epitopes, including antibodies with cross-neutralizing activity across related flaviviruses. In particular, studies of human ZIKV and DENV infections demonstrate the emergence of recurrent antibody lineages targeting conserved regions of the envelope protein, providing insight into how immune history and epitope targeting influence antibody breadth [76,112]. These observations support a conceptual framework in which VLP immunogens that faithfully present conserved envelope surfaces could preferentially recall or shape such memory B-cell responses, thereby promoting durable and cross-reactive humoral immunity.

Although VLPs vaccines are a promising tool, some challenges need to be overcome before they reach clinical application. In the flavivirus context, VLPs are often weak immunogens, and they require several injections or supplementation with strong adjuvants to reach sufficient protection. For instance, a 100% protection by a JEV-VLPs-based vaccine in BALB/c mice could only be reached when the vaccine was linked to an adjuvant. In the absence of an adjuvant, protection was only 40% [209].

Two important factors to consider when producing a flavivirus VLP vaccine are size and maturation of the VLPs. Ohaki and colleagues demonstrated that large (40–50 nm) mature VLPs (similar size as natural virions) induced higher NT-Ab-neutralizing antibody titers than smaller (20–30 nm), immature VLPs. In addition, the large and mature VLPs exhibited more potent protection against WNV in challenged C3H/HeN mice [210]. The production of larger, mature, and more efficient VLPs could be influenced by several hard-to-manage and flavivirus-specific factors, such as N-linked glycosylation of the E protein, which affects the processing and folding of the protein [211].

Another factor to consider is that despite their intrinsic advantages, early-generation flavivirus VLPs often exhibited heterogeneity in maturation and E-protein conformation. Recent work has addressed this limitation through structure-guided VLP engineering, particularly targeting the fusion loop and E-dimer interface. A notable example is the stabilization of the E proteins via targeted mutations. Introduction of a cysteine substitution at the E dimer interface (A264C) locked E proteins in their pre-fusion dimeric conformation, preserving quaternary neutralizing epitopes while suppressing fusion loop exposure [48]. VLPs incorporating this modification elicited potent neutralizing antibodies and conferred protection in vivo, highlighting the importance of conformational control in VLP design.

Cellular debris from the cell line used to produce the VLPs could also cross-react and inhibit immunogenic stimulus on B-cell clones at high doses [210]. ADE is another challenge in the design of effective VLPs for flavivirus vaccines. Care needs to be taken to avoid the inclusion of problematic ADE-prone regions [198], and direct the mutation on the fusion loop [201]. Some groups opt to co-express several quaternary epitopes from the E protein in the same formulation, which enabled the development of a DENV “four-in-one” VLP vaccine that protected immunized AG129 mice from DENV1-4 infections [212].

### 5.3. Potential of mRNA-Encoded Multivalent Antigens

The success of SARS-CoV-2 mRNA vaccines [213] has catalyzed mRNA platforms to encode flavivirus immunogens, including quaternary epitope-stabilized constructs. mRNA vaccines offer multiple advantages: rapid scalability, non-infectious, and host processing of the antigenic protein (e.g., E dimers), resembling a natural infection [214].

Recently, 3xEIII, an mRNA vaccine for ZIKV encoding a triple repeat of DIII, elicited neutralizing antibodies, effectively eliminating the virus from the organs of challenged C57BL/6 mice and conferring protection and long-term immunity. Long-term immunity was characterized by an increase in cytokine production, such as IL-4, and activation of T cell memory markers. Analysis of long-term immune responses revealed sustained antibody levels up to 40 weeks post-immunization [215].

Durably elevated antibody titers are typically limited in conventional mRNA vaccines, likely due to several reasons, such as waning plasma cells, as seen with current mRNA vaccines (e.g., SARS-CoV-2 [216]) or the appearance of new variants of the virus [217]. To address this, Lu and colleagues generated a self-amplifying RNA (saRNA) vaccine encoding the mature membrane and E proteins from ZIKV. C57BL/6 mice were immunized and administered two agonist antibodies to promote effector and memory T cell responses. They showed that a single dose of the antibodies, administered on consecutive days, markedly boosted the production of ZIKV-specific polyfunctional and cytotoxic CD8+ T cell responses, decreasing viral load by 2-log units compared to untreated mice [218].

Early-phase human trials of saRNA vaccines have demonstrated favorable safety profiles, though transient local and systemic adverse events were common. Participants of a Phase I/II study of ARCT-021, a COVID-19 saRNA vaccine, experienced dose-dependent adverse reactions, including injection site pain, fatigue, headache, and fever [219]. Similarly, recent clinical data from two studies of saRNA vaccines, one for rabies and another for cancer, have also shown low toxicity, with most adverse events being mild or moderate [220,221]. While these findings are encouraging, long-term safety data are limited, and further studies are needed to fully characterize the risk profile across different populations.

Another promising area of development in flavivirus vaccine design is the decrease in undesirable side effects, such as ADE. mRNA vaccines that display epitope modification to enhance the immune response can avoid ADE and enable the generation of bNAbs. Kumari and colleagues used mRNA-containing lipid nanoparticles, carrying a modified DENV2 E protein, to immunize BALB/c mice. The mice elicited high antibody titers and enhanced neutralizing activity, along with a reduced ADE burden. The E protein mutation consisted of an amino acid substitution in residue N8 of the fusion loop. Moreover, the group is currently enhancing the stability and protein expression of the mRNA molecule by adding untranslated regions (UTR) to both sides of the gene sequence. Prospective studies aim to evaluate the neutralizing activities of immunized-mouse sera against DENV1, 3, and 4 to assess neutralization of all DENV serotypes [222].

Since ADE activity is a remaining concern in developing flavivirus vaccines, efforts are being made to reduce the ADE burden. JEV-E^mut^ mRNA-LNP is an mRNA-based vaccine that elicited the production of neutralizing antibodies in C57BL/6 mice, immunizing and protecting them from mortality by JEV infection. Furthermore, passively transferred sera from vaccinated animals did not lead to obvious ADE of ZIKV in recipient mice. Likewise, this study highlights the role of optimizing the FL sequence, using amino acid substitutions in the fusion loop sequence of JEV to reduce the level of cross-reactive non-neutralizing antibodies with other flaviviruses, such as ZIKV [223].

Collectively, these strategies converge on the goal to stabilize and present dimeric-quaternary epitopes in their native conformation (as opposed to monomeric immunogens) as a safe and effective target in developing a pan-flavivirus vaccine, overcoming the ADE, providing durable protection, and enhancing immunogenicity (Table 2).

### 5.4. Addressing ADE in Pan-Flavivirus Vaccine Development

A central conceptual concern in developing pan-flavivirus vaccines is ADE, a phenomenon in which non-neutralizing or sub-neutralizing antibodies facilitate increased viral entry into Fcγ receptor–bearing cells and exacerbate disease severity. ADE has been most extensively documented in DENV immunology, where antibodies from a primary infection can enhance infection by a different DENV serotype, increasing the risk of severe disease during secondary exposure [182]. Cross-reactive antibodies elicited by infection or vaccination that bind but do not efficiently neutralize heterologous flaviviruses can contribute to ADE in vitro and in vivo [28,225].

Importantly, the immunodominance of certain E protein regions—notably the fusion loop in DII—has been implicated in ADE because antibodies targeting these conserved regions readily cross-react among flaviviruses but often lack potent breadth and neutralization potency. This has raised legitimate safety concerns for pan-flavivirus immunogens that include highly conserved but poorly neutralizing epitopes [226].

Structural vaccinology offers strategies to address these ADE risks by reshaping antigenic surfaces to bias the immune response toward protective, conserved quaternary epitopes rather than enhancing ones. For example:

− Epitope resurfacing and epitope masking approaches can selectively occlude immunodominant fusion loop epitopes that are associated with enhancement, while preserving or enhancing presentation of potent neutralizing quaternary determinants (e.g., E dimer–dependent epitopes). This strategy has been explored in engineered DENV immunogens that redirect antibody responses away from enhancement-linked regions [88].
− Stabilization of native pre-fusion envelope structures (e.g., via engineered disulfide bonds or stabilizing mutations at the dimer interface) increases the fidelity of quaternary epitope presentation and reduces exposure of cryptic enhancing epitopes. Such stabilization can improve both neutralization potency and breadth while reducing binding to poorly neutralizing, cross-reactive surfaces [183].

On the other hand, animal challenge studies offer an important window into ADE risk in pre-clinical models:

− In macaque models, prior exposure to DENV followed by ZIKV challenge has been used to explore whether cross-reactive antibodies can enhance disease. Several studies have found that highly neutralizing, affinity-matured antibody responses did not lead to increased disease, whereas sub-neutralizing antibody levels correlated with increased viral load in target tissues in some settings [227].
− In mouse models, passive transfer of poorly neutralizing DENV antibodies exacerbated infection, whereas transfer of well-characterized, potently neutralizing monoclonal antibodies did not result in enhancement and instead conferred protection, supporting the notion that the quality and specificity of the antibody response—not merely its presence—determines ADE outcomes [228].
− Flavivirus VLP immunization studies in small animals have shown that VLPs engineered to remove or mask fusion loop epitopes elicit balanced, high-titer neutralizing responses without signs of enhancement upon challenge with homologous or heterologous viruses, providing proof-of-concept that antigen design can mitigate ADE risk [229].

Taken together, these findings underscore that ADE is a significant, mechanistically understood phenomenon that cannot be ignored in pan-flavivirus vaccine development. Structural vaccinology, combined with protein engineering and epitope selection strategies, provides a credible and evidence-based path forward for the development of pan-flavivirus vaccines that elicit broad neutralization without promoting enhancement.

## 6. Conclusions and Future Directions

Looking ahead, novel strategies such as delivering disulfide-stabilized E dimers via mRNA and assembling modified quaternary or multivalent mRNA epitopes in VLPs could lead to a new generation of rationally designed flavivirus vaccines. These tools not only offer enhanced immunogenicity but also reduce the ADE risk, fulfilling the need for safe, effective, and long-lasting protection against flavivirus.

Although the antibody response has been the focus of the immunological response, the cellular immune response is gaining attention as lessons from DENV and ZIKV trials have demonstrated T-cell immunity as an essential component of safe, efficacious, and durable flavivirus vaccines [230]. Hence, defining proper correlates of protection, both humoral and cellular [231], is key to the development of an effective pan-flaviviruses vaccine.

Multi-epitope vaccine platforms are being explored to improve the engagement of T cell responses. New research is highlighting the need to include nonstructural proteins (particularly, NS3 and NS5) in the vaccine formulation for the induction of a CD8+ T-cell response [232].

Additionally, other studies have described how immune effector cells respond to specific viral components. For instance, CD4+ T cells and B cells are skewed toward the recognition of viral components, such as envelope, capsid, and NS1, whereas CD8+ T cells preferentially target nonstructural proteins (NS3 and NS5) [232]. Furthermore, analyses of DENV infections indicate that robust responses by multifunctional CD8+ T cells are correlated with protection against severe disease [233].

Modern technologies like nuclear magnetic resonance spectroscopy [62] and X-ray crystallography [76] have revealed highly conserved regions among flaviviruses’ sequences. However, divergence has resulted in different species expressing distinct antigenic profiles, requiring careful consideration of immunogens in vaccine development strategies. For instance, the E protein from YFV exhibits the least sequence similarity with the other mosquito-borne flaviviruses at approximately 40–66%. In fact, its E protein is more closely related to YFV’s distant, tick-borne relatives, such as the Wesselsbron virus [234].

Other flaviviruses, such as JEV and WNV, share greater similarity in the E protein sequence (approximately 77%) [234]. Identifying these structural and antigenic variations is paramount in the design of a pan-flavivirus vaccine. New strategies, such as machine learning-assisted phylogenetics modeling, are now being used, which could provide a more comprehensive map of overlapping regions within the many flavivirus genomes, enabling the identification of targets in the search for a broad-spectrum vaccine [235].

In conclusion, the dual challenges of ADE and variable epitope targeting call for a shift toward rational vaccine design strategies that prioritize safety, specificity, durable immunity, and broad protection. By re-engineering immunodominant antigenic regions, selectively including protective, quaternary epitopes, and engaging both humoral and cellular immune responses, a pan-flavivirus vaccine holds the potential to overcome limitations of earlier species-specific candidates.

## Figures and Tables

**Table 2:** Monomeric vs. Dimeric Immunogens in Experimental Flavivirus Vaccines.

Attribute	Monomeric E Immunogen	Dimeric (or Stabilized Dimer) E Immunogen
Structural Presentation	Monomeric versions of DENV2 E protein elicited non-neutralizing, cross-reactive antibodies, potentially prone to ADE activity [170,184].	Stable aligned dimers mimicking native virion curvature [183,187]. Stabilized E-dimers actively avoiding expression of the fusion loop and adjacent regions to overcome ADE [198]
	Monomeric immunogens often include fusion loop regions and prM proteins, strongly associated with ADE [28,183,196]	
Quaternary Epitope Display	Poor-quaternary epitopes and/or often largely absent [182,183]	Locking E-dimer in a native-like conformation by inter-subunit disulfide bonds, reducing exposure of non-neutralizing immunodominant regions [183,196]
Recognition by Neutralizing mAbs	Limited, often fusion loop-focused, poorly neutralizing [148,190]	Specific human bnAbs protect against DENV1-4, JEV, WNV, and ZIKV [88,179,184]
Immune Response Quality	Often biased toward fusion loop and type-specific responses, which could potentially lead to ADE [47,188]	Broader, stronger neutralizing responses, including cross-type [70,148]
Neutralizing Antibody Titers	Low to moderate, susceptible to ADE risks [10]	Higher titers, improved specificity [70,148]: see Table 1
Production/Stability	Easier to produce [151]	Requires engineering (for instance, disulfide bonds or mutation sets) but yields stabilized dimers with improved thermostability and expression [177,178,179,185]
Pan-Flavivirus Potential	Limited cross-protection [150,171]	High potential when engineered to expose cross-reactive quaternary epitopes [149,224].

## Data Availability

No new data were created or analyzed in this study. Data sharing is not applicable to this article.
